# Protein phosphatase 2A-B55β mediated mitochondrial p-GPX4 dephosphorylation promoted sorafenib-induced ferroptosis in hepatocellular carcinoma via regulating p53 retrograde signaling

**DOI:** 10.7150/thno.82132

**Published:** 2023-07-31

**Authors:** Bo Qian, Lin Che, Ze-Bang Du, Ni-Jun Guo, Xin-Mou Wu, Lei Yang, Zhao-Xuan Zheng, Yun-Lu Gao, Ming-Zhu Wang, Xiao-Xuan Chen, Ling Xu, Zi-Jian Zhou, Yu-Chun Lin, Zhong-Ning Lin

**Affiliations:** 1 State Key Laboratory of Vaccines for Infectious Diseases, Xiang An Biomedicine Laboratory; State Key Laboratory of Molecular Vaccinology and Molecular Diagnostics; National Innovation Platform for Industry-Education Integration in Vaccine Research; School of Public Health, Xiamen University, Xiamen, China.; 2 Center for Molecular Imaging and Translational Medicine, School of Public Health, Xiamen University, Xiamen, China.

**Keywords:** Ferroptosis, Glutathione peroxidase 4, Retrograding p53 signal, Protein phosphatase 2A-B55β subunit, Hepatocellular carcinoma

## Abstract

**Rationale:** As a key endogenous negative regulator of ferroptosis, glutathione peroxidase 4 (GPX4) can regulate its antioxidant function through multiple post-translational modification pathways. However, the effects of the phosphorylation/dephosphorylation status of GPX4 on the regulation of inducible ferroptosis in hepatocellular carcinoma (HCC) remain unclear.

**Methods:** To investigate the effects and molecular mechanism of GPX4 phosphorylation/dephosphorylation modification on ferroptosis in HCC cells. Sorafenib (Sora) was used to establish the ferroptosis model in HCC cells *in vitro*. Using the site-directed mutagenesis method, we generated the mimic GPX4 phosphorylation or dephosphorylation HCC cell lines at specific serine sites of GPX4. The effects of GPX4 phosphorylation/dephosphorylation modification on ferroptosis in HCC cells were examined. The interrelationships among GPX4, p53, and protein phosphatase 2A-B55β subunit (PP2A-B55β) were also explored. To explore the synergistic anti-tumor effects of PP2A activation on Sora-administered HCC, we established PP2A-B55β overexpression xenograft tumors in a nude mice model *in vivo*.

**Results:** In the Sora-induced ferroptosis model of HCC *in vitro*, decreased levels of cytoplasmic and mitochondrial GPX4, mitochondrial dysfunction, and enhanced p53 retrograde signaling occurred under Sora treatment. Further, we found that mitochondrial p53 retrograded remarkably into the nucleus and aggravated Sora-induced ferroptosis. The phosphorylation status of GPX4 at the serine 2 site (GPX4^Ser2^) revealed that mitochondrial p-GPX4^Ser2^ dephosphorylation was positively associated with ferroptosis, and the mechanism might be related to mitochondrial p53 retrograding into the nucleus. In HCC cells overexpressing PP2A-B55β, it was found that PP2A-B55β directly interacted with mitochondrial GPX4 and promoted Sora-induced ferroptosis in HCC. Further, PP2A-B55β reduced the interaction between mitochondrial GPX4 and p53, leading to mitochondrial p53 retrograding into the nucleus. Moreover, it was confirmed that PP2A-B55β enhanced the ferroptosis-mediated tumor growth inhibition and mitochondrial p53 retrograde signaling in the Sora-treated HCC xenograft tumors.

**Conclusion:** Our data uncovered that the PP2A-B55β/p-GPX4^Ser2^/p53 axis was a novel regulatory pathway of Sora-induced ferroptosis. Mitochondrial p-GPX4^Ser2^ dephosphorylation triggered ferroptosis via inducing mitochondrial p53 retrograding into the nucleus, and PP2A-B55β was an upstream signal modulator responsible for mitochondrial p-GPX4^Ser2^ dephosphorylation. Our findings might serve as a potential theranostic strategy to enhance the efficacy of Sora in HCC treatment through the targeted intervention of p-GPX4 dephosphorylation via PP2A-B55β activation.

## Introduction

Hepatocellular carcinoma (HCC) is one of the most common causes of cancer-related death globally 1. Sorafenib (Sora) is one of the medically-approved systemic treatments that has demonstrated a survival benefit in patients with unresectable HCC 2. However, some HCC patients are of limited effectiveness to Sora, and some patients acquire drug resistance 2. Ferroptosis is a new approach for HCC treatment characterized by the accumulation of toxic lipid peroxidation (LPO) due to the loss of glutathione peroxidase 4 (GPX4) 3. GPX4 is the only enzyme that can diminish lipid hydroperoxides of biological membranes and plays a primary role in regulating cells that undergo ferroptosis 4. The anti-cancer effects of Sora may be partially explained by its ability to induce ferroptosis via interfering with the antioxidant function of GPX4 5. GPX4 seems to be particularly relevant in cancer development. The overall survival of glioma patients with low GPX4 expression was longer than that of GPX4 high expression group 3, 6.

Moreover, the inhibition of GPX4 induces growth suppression of tumors, especially for drug-resistance tumors 7, 8. Combining androgen-deprivation therapy drugs with GPX4 inhibitors has achieved a remarkable synergistic effect on resistant prostate cancer treatment 9. Therefore, targeting GPX4 inhibition might be a potential approach to improve the susceptibility of HCC to Sora treatment through enhancing ferroptosis. The current approach for GPX4 inhibition is achieved using a pharmacological method. The existing GPX4 inhibitors are covalently reacted with GPX4 through the reactive alkyl chloride, giving poor selectivity and pharmacokinetics 10. It is thus imperative to find an effective intervention approach for targeting GPX4 to improve the anti-cancer effects via ferroptosis induction.

The different subcellular locations of GPX4 bring out its distinct roles. Among them, mitochondrial GPX4 was more efficient than non-mitochondrial GPX4 in protecting cells from mitochondria damage and extracellular oxidative stress (such as t-butyl-hydroperoxide, photosensitizers, H_2_O_2_, and 15-hydroperoxy-eicosatetraenoic acid) 11, 12. Despite the role of non-mitochondrial GPX4 in ferroptosis being clearly defined 13, the underlying molecular mechanisms of mitochondrial GPX4 in ferroptosis remain unclear. p53 was involved in the GPX4 modulation via multiple pathways 14, 15, whereas the relationship and crosstalk between mitochondrial GPX4 and p53 are still missing. Mitochondrial p53 alterations might be the upstream signals for Sora-induced ferroptosis. Sora-induced mitochondrial translocation of p53 in the hepatic stellate cells triggered ferroptosis through direct interaction with the solute carrier family 25 member 28 16. Moreover, the translocation of p53 from the mitochondria to the nucleus, called the p53 retrograde signaling, is also an early signal of mitochondrial dysfunction 17. Given the vital role of mitochondrial p53 in regulating mitochondria homeostasis and mitochondria-related ferroptosis, it is reasonable to speculate that mitochondrial p53 alterations might be involved in mitochondrial GPX4-regulated ferroptosis.

Evidence recently revealed that post-translational modification (PTM) of GPX4 might be a new strategy for ferroptosis modulation. Ding et al. pointed out that the ubiquitination of GPX4 triggered by DMOCPTL (a derivative of the natural product parthenolide) could induce triple-negative breast cancer cell ferroptosis 18. Moreover, the mechanism of RSL3-induced ferroptosis was evidenced to be associated with the amino acid modification of GPX4 in the active region 19. The online prediction software suggested that serine (Ser)/threonine (Thr) residues in GPX4 might be potential phosphorylation/dephosphorylation modification targets. Therefore, exploring the role of GPX4 phosphorylation/dephosphorylation modification in ferroptosis might provide an attractive intervention strategy for ferroptosis-associated cancer treatment.

The present study revealed that the dephosphorylation of mitochondrial p-GPX4^Ser2^ participated in Sora-induced ferroptosis in HCC by inducing mitochondrial p53 translocation into the nucleus and PP2A-B55β (encoded by the *PPP2R2B* gene) was involved in p-GPX4^Ser2^ dephosphorylation. As a result, the combination using of the PP2A-B55β activator and Sora is a novel theranostic approach for HCC treatment.

## Results

### Ferroptosis resistance and high GPX4 expression were associated with HCC

**Figure 1:**
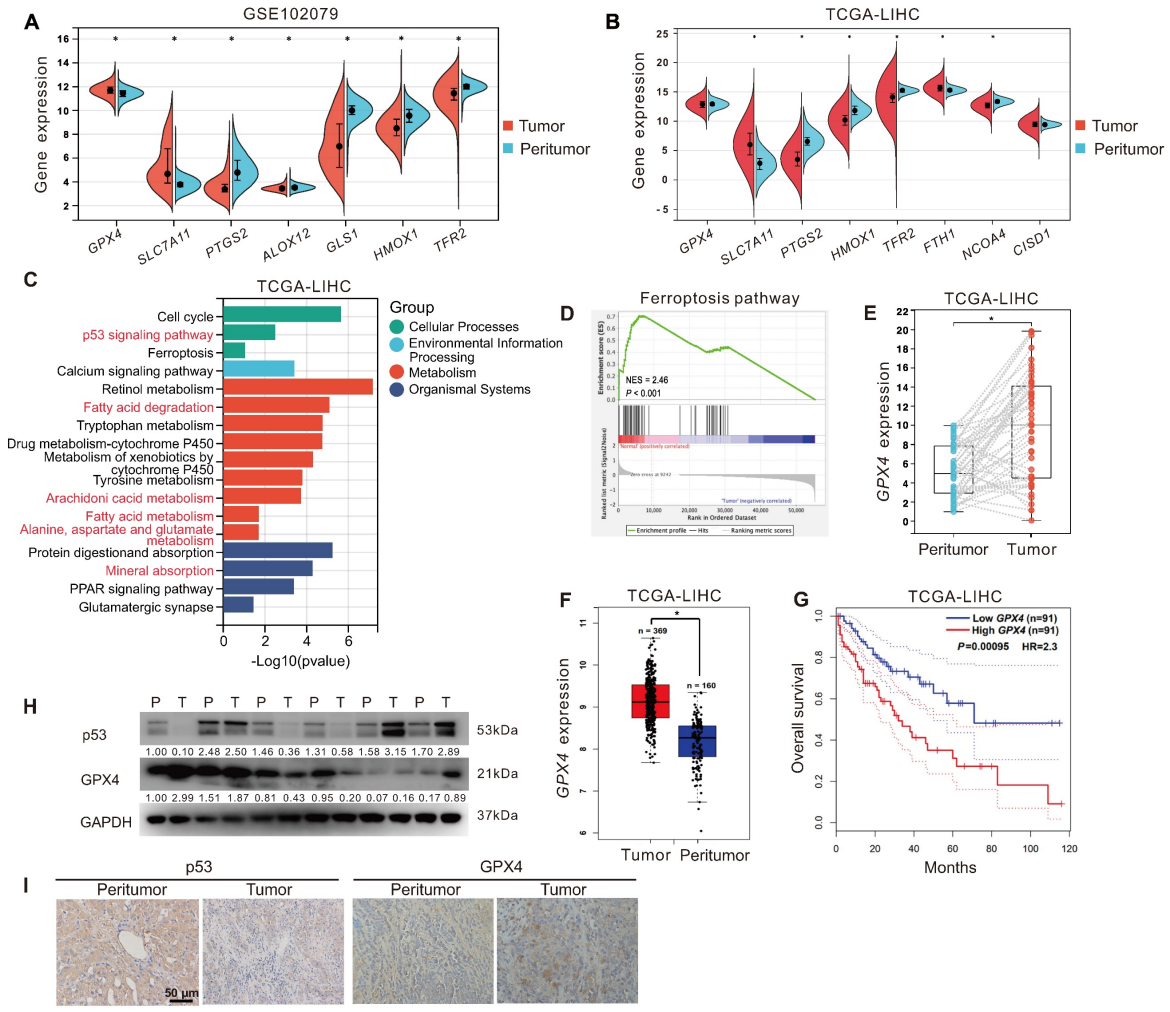
** Ferroptosis resistance and high GPX4 expression were associated with HCC. A.** The expression of ferroptosis-related genes in tumor tissues (n = 152) and peritumor tissues (n = 91) of HCC patients from the GEO database (GSE102079). **B.** The expression of ferroptosis-related genes in tumor tissues (n = 373) and peritumor tissues (n = 50) of HCC patients from the TCGA-LIHC database.** C.** KEGG analysis of DEGs between tumor tissues and peritumor tissues in HCC patients from TCGA-LIHC database. **D.** GSEA analysis of the ferroptosis pathways in tumor tissues and peritumor tissues of HCC patients from TCGA-LIHC database. **E.** *GPX4* expression in the paired tumor tissues (n = 40) and the paired peritumor tissues (n = 40) from the TCGA-LIHC database. **F.** *GPX4* expression in two cohorts of HCC patients from GEPIA. **G.** Kaplan-Meier analysis showed the overall survival of HCC patients from GEPIA (TCGA-LIHC) with different levels of *GPX4* expression. *GPX4* expression was a binary variable divided into high or low expression according to the quartile. **H.** Protein levels of p53 and GPX4 in the paired peritumor tissues (P) and the paired tumor tissues (T) from the HCC patients, n = 6. **I**. Representative IHC images of p53 and GPX4 in the paired peritumor tissues and paired tumor tissues from the HCC patients. *, *P* < 0.05.

To explore the relationship between ferroptosis and HCC development. First, we analyzed the expression of ferroptosis-related genes in tumor tissues (n = 152) and peritumor tissues (n = 91) of HCC patients from the GEO dataset (GSE102079). As displayed in Figure 1A, compared to that in peritumor tissues, the expression of ferroptosis-inducing genes, including *PTGS2*, *ALOX12*,* TFR2,* and* HMOX1,* was decreased in tumor tissues, whereas ferroptosis-suppressor genes, including* SLC7A11* and *GPX4*, were increased. Next, we analyzed the expression of ferroptosis-related genes in tumor tissues (n = 373) and peritumor tissues (n = 50) of HCC patients from the TCGA-LIHC dataset. Similarly, compared to that in peritumor tissues, the expression of ferroptosis-inducing genes, including* PTGS2*,* HMOX1, TFR2*, and *NCOA4*, was down-regulated in tumor tissues, whereas ferroptosis-suppressor genes* SLC7A11* and* FTH1* were upregulated (Figure 1B). KEGG analysis showed that the DEGs between tumor tissues and peritumor tissues of HCC patients from the TCGA-LIHC dataset were enriched in several ferroptosis-related pathways, such as p53 signaling pathway, fatty acid metabolism, fatty acid degradation, arachidonic acid metabolism, and alanine, aspartate, and glutamate metabolism (Figure 1C). Furthermore, GSEA analysis showed that the ferroptosis pathway was upregulated in peritumor tissues but not in tumor tissues of HCC patients from the TCGA-LIHC dataset (Figure 1D, normalized enrichment scores = 2.46, *P* < 0.05). The evidence above suggested that ferroptosis resistance was associated with the development of HCC.

As GPX4 is a crucial endogenous negative regulator of ferroptosis, we analyzed the expression of GPX4 in 40 tumor tissues and 40 paired peritumor tissues of HCC patients from the TCGA-LIHC dataset. The results showed that the expression of *GPX4* in tumor tissues was upregulated compared to in peritumor tissues (*P < 0.05*, Figure 1E). Besides, we further analyzed the correlation between* GPX4* and HCC prognosis in HCC patients from the GEPIA database. Compared to that in peritumor tissues (n = 160), the expression of *GPX4* gene was significantly increased in tumor tissues (n = 369) of HCC patients (Figure 1F). As shown in Figure 1G, compared to the HCC patients with higher *GPX4* mRNA expression (n = 91), HCC patients with lower *GPX4* mRNA expression (n = 91) had a higher overall survival rate (*P < 0.05*), indicating that GPX4 was negatively associated with HCC prognosis. Additionally, compared to that in peritumor tissues, the protein level of GPX4 was increased in the majority (2/3) of tumor tissues from HCC patients, whereas the protein level of p53 was decreased in half (1/2) of tumor tissues from HCC patients (Figure 1H-I). The above results indicated that resistance to ferroptosis and high expression of GPX4 were associated with the development of HCC.

### Downregulation of GPX4 and mitochondrial dysfunction in Sora-induced ferroptosis of HCC cells

**Figure 2:**
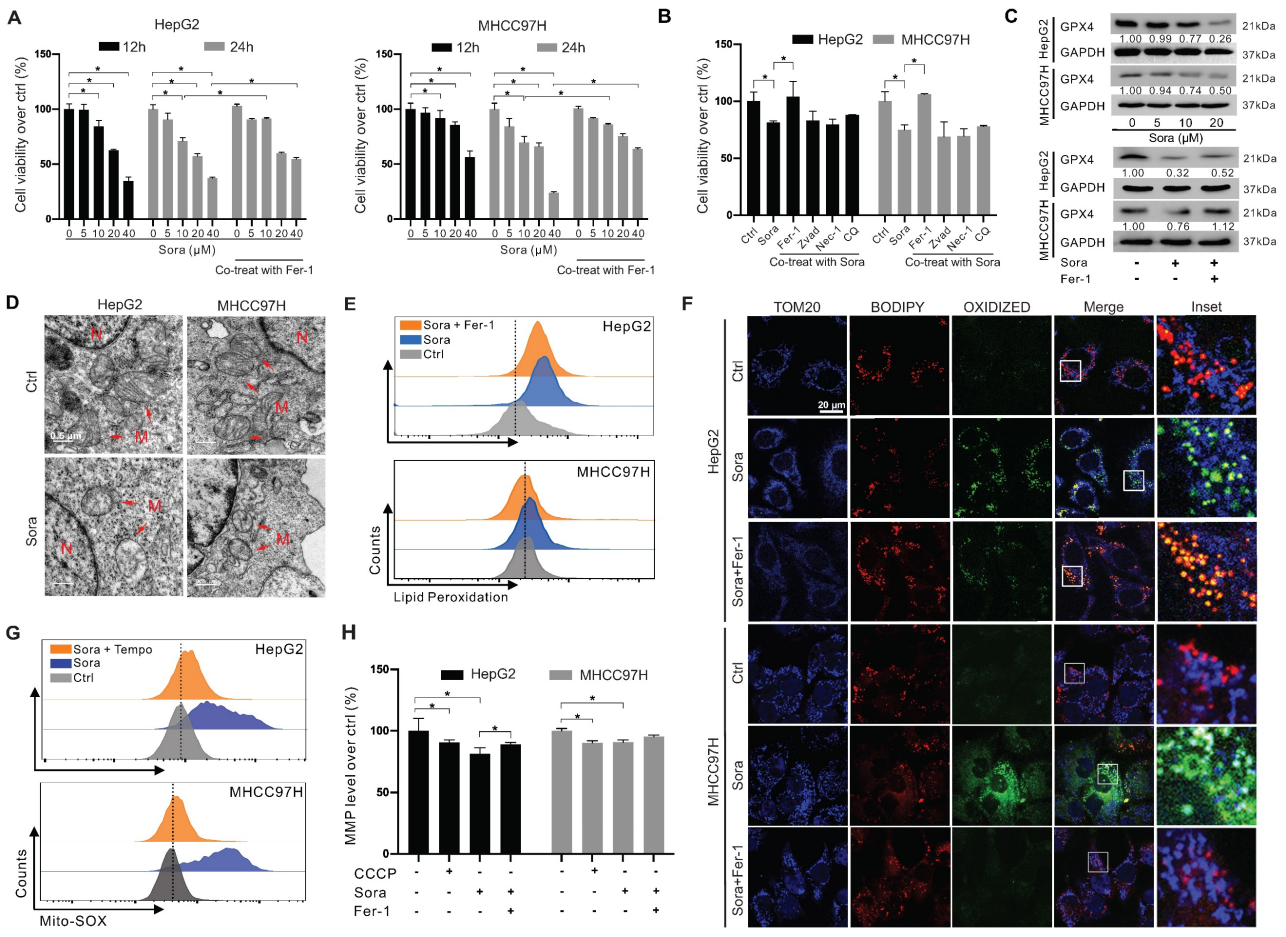
** Downregulation of GPX4 and mitochondrial dysfunction in sorafenib-induced ferroptosis of HCC cells. A.** HCC cells were treated with different doses of sorafenib (Sora, 5, 10, 20, and 40 µM, for 12 h and 24 h) alone or combined with ferroptosis inhibitor Fer-1 (1 µM) for 24 h. Cell viability was measured with the MTS assay. **B.** HCC cells were treated with Sora (10 µM) individually or combined with inhibitors of different cell death patterns, including Fer-1 (1 µM), Z-VAD (10 µM), Nec-1 (10 µM), or CQ (5 µM) for 24 h. Cell viability was measured with the MTS assay. **C.** WB detection of GPX4 protein expression in HCC cells after treatment with different doses of Sora (5, 10, 20 µM, 24 h) alone or combined with Fer-1 (1 µM, 24 h). **D.** TEM observation of mitochondrial morphological characteristics (red arrows) of ferroptosis in HCC cells treated with Sora (10 µM, 24 h). N, nucleus; M, mitochondria. Scale bar, 0.5 μm.** E.** FCM detection of LPO levels in HCC cells treated with Sora (10 μM, 24 h) individual or combined with Fer-1 (1 μM, 24 h).** F.** IF detection of mitochondrial LPO in HCC cells treated with Sora (10 µM, 24 h). Scale bars, 20 μm. **G.** FCM detection of mitochondrial ROS in HCC cells treated with Sora (10 µM, 24 h) individual or combined with Mito-Tempo (20 µM, pretreatment for 2 h). **H.** FCM detection of mitochondrial membrane potential in HCC cells treated with CCCP (10 µM, 6 h) and Sora (10 µM, 24 h) individual or combined with Fer-1 (1 μM, 24 h) in HCC cells. *, *P* < 0.05.

Sora was reported to exert anti-tumor effects on HCC via inducing ferroptosis 20. We next explored the role of GPX4 in Sora-induced ferroptosis. As shown in Figure 2A, the cell viability of HCC cells decreased in a dose-dependent manner of Sora treatment, while the Sora-induced cell death could be rescued by ferroptosis inhibitor Fer-1. However, in contrast to that under the low-dose Sora (10 µM), Sora-induced cell death cannot be rescued to near-normal levels by Fer-1 under high-dose conditions (20 or 40 µM) (Figure 2A), suggesting that high-doses Sora may reduce the ferroptosis sensitivity of HCC cells. Low-dose Sora was evidenced to be more efficient and safer than high-dose Sora in NASH-induced HCC of mice 20. Using of 10 μM Sora in the following experiments was based on the pharmacokinetic analysis of Sora in the plasma (9.7-12 µM) of patients with HCC 21. As shown in Figure 2B and Figure S1A, Sora-induced cell death cannot be rescued by the apoptosis inhibitor (Zvad), necroptosis inhibitor (Nec-1), or autophagy inhibitor (chloroquine, CQ) (Figure 2B), but rescued by the co-treatment of Fer-1, indicating that Sora specifically induced ferroptosis in HCC. Next, the GPX4 level was measured in Sora-treated HCC cells. As expected, Sora treatment decreased the protein level of GPX4 in HCC cells, and the changes were alleviated with Fer-1 co-treatment (Figure 2C). Also, we examined the effects of Sora on system Xc- mediated GSH synthesis and iron metabolism in HCC cells. As shown in Figure S1B-D, the expression of the solute carrier family 7 member 11 (SLC7A11) subunit of system Xc- and the level of GSH were decreased in Sora-treated HCC cells, whereas there were no significant changes observed in the expression of transferrin receptor (TFRC), Fe^3+^ reductase six-transmembrane epithelial antigen of prostate 3 (STEAP3), ferritin heavy chain 1 (FTH1), and the iron-pumping protein ferroportin 1 (FPN1) in Sora-treated HCC cells. These findings demonstrated that Sora induced GPX4-related ferroptosis in HCC cells.

It was reported that GPX4 regulated ferroptosis by controlling mitochondrial function 22. We explored the mitochondrial function in Sora-treated HCC. As shown in Figure 2D, Sora-treated HCC cells presented a decrease in the mitochondrial cristae (one of the ferroptosis markers) and an increase in the electron density of the outer mitochondrial membrane (OMM) using the TEM technique. Ferroptosis is accompanied by the massive generation of LPO 22. FCM analysis showed that Sora induced obvious LPO accumulation in HCC cells (Figure 2E). With IF staining, the oxidized lipid (green dots) and its overlay (cyan dots) with the OMM protein TOM20 (blue dots) were increased in Sora-treated HCC cells (Figure 2F), suggesting that Sora induced LPO accumulation in mitochondria of HCC cells. Moreover, cellular ROS (Figure S1E) and mitochondrial ROS (Figure 2G) were increased in HCC cells under Sora treatment. Additionally, consistent with the change in CCCP (a positive inducer of mitochondrial membrane damage) treated cells, Sora decreased the mitochondrial membrane potential (MMP) level of HCC cells (Figure 2H). These Sora-driven mitochondrial changes were alleviated by Fer-1 co-treatment. These results indicated that mitochondrial dysfunction was associated with Sora-induced ferroptosis in HCC cells.

### Mitochondrial p53 retrograde into the nucleus was involved in Sora-induced ferroptosis

**Figure 3:**
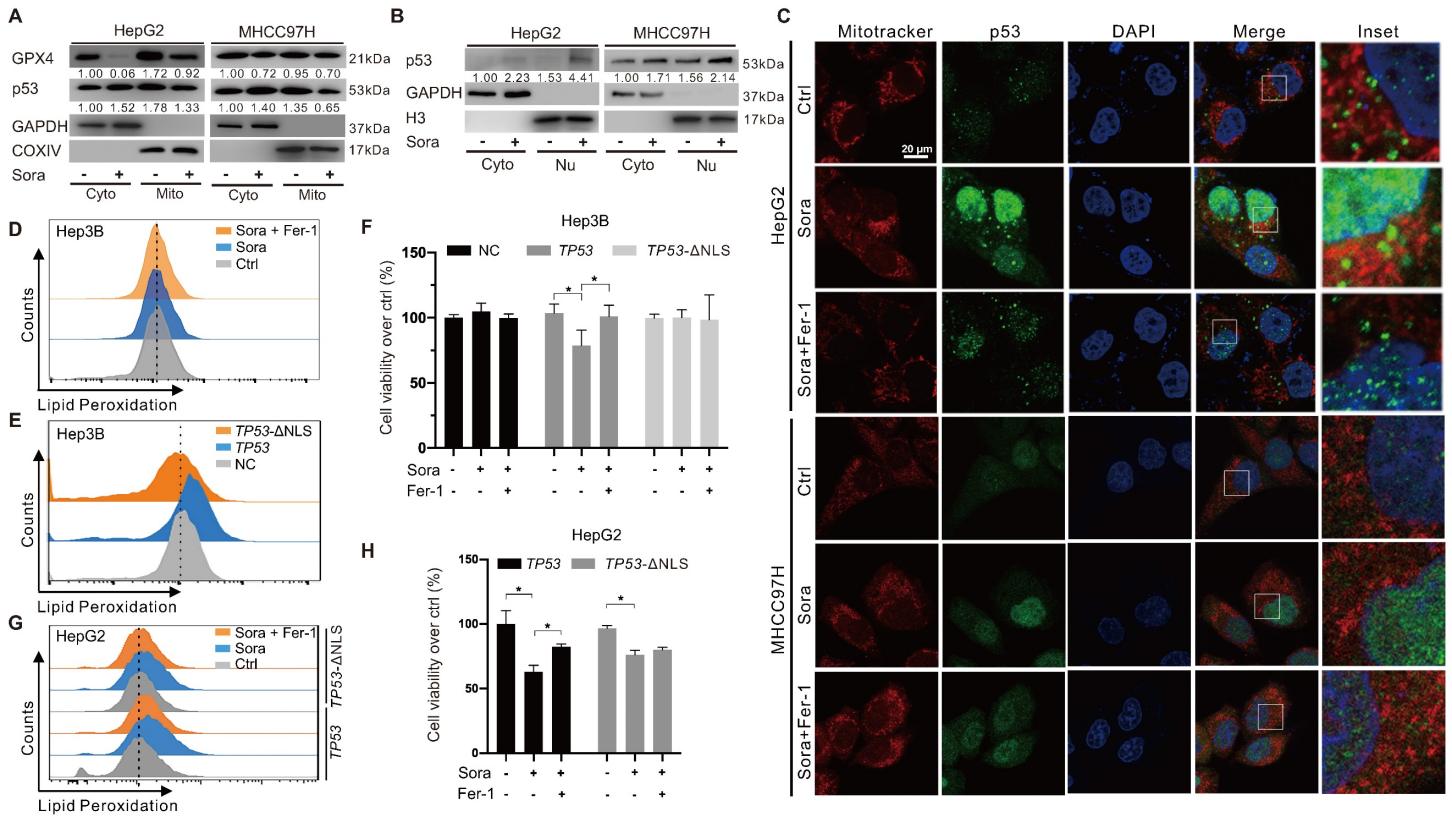
** Mitochondrial p53 retrograde into the nucleus was involved in Sora-induced ferroptosis. A.** Protein levels of p53 in cytosolic (Cyto) or mitochondrial (Mito) fraction of HCC cells treated with Sora (10 µM, 24 h). **B.** Protein levels of p53 in cytosolic (Cyto) or nucleus (Nu) fraction of HCC cells treated with Sora (10 µM, 24 h). **C.** IF assay of the p53 distribution in mitochondria and nucleus of HCC cells treated with Sora individual (10 µM, 24 h) or combined with Fer-1 (1 µM, 24 h). Cells were subjected to IF staining of p53 (green), Mitotracker (red), and DAPI (blue). Scale bars, 20 μm. **D.** FCM detection of LPO in Hep3B cells. Staining cells with C11 BODIPY probe. **E.** FCM detection of LPO in Hep3B cells transfected with a p53-expressing plasmid or p53 nuclear localization sequence deleted (ΔNLS) plasmid. Staining cells with C11 BODIPY probe. **F.** Hep3B cells transfected with a p53-expressing plasmid or p53 ΔNLS plasmid were treated with Sora (10 µM, 24 h) individually or combined with Fer-1 (1 µM, 24 h). Cell viability was measured with the MTS assay. **G-H.** HepG2 cells transfected with a p53-expressing plasmid or p53 ΔNLS plasmid were treated individually with Sora (10 µM, 24 h) or combined with Fer-1 (1 µM, 24 h). Staining cells with a C11 BODIPY probe and LPO levels were measured with the FCM assay (G). Cell viability was measured with the MTS assay (H). *, *P* < 0.05.

The retrograde signal of p53 was reported as an early event of mitochondrial dysfunction 17. We investigated whether mitochondrial p53 translocation was involved in Sora-induced mitochondrial dysfunction and ferroptosis. Along with the decline in mitochondrial GPX4, diminished mitochondrial p53 and elevated nucleus p53 were observed in Sora-treated HCC cells (Figure 3A-B). Consistently, Sora treatment attenuated p53 (green) localization in mitochondria (red), whereas it enhanced the p53 (green) signal in the nucleus (blue) (Figure 3C), further revealing that mitochondrial p53 retrograding into the nucleus occurred under Sora treatment.

Hep3B cells with a null-p53 background were selected for the subsequent studies to determine whether p53 retrograding into the nucleus affected the occurrence of ferroptosis. As shown in Figure 3D, Sora treatment did not alter LPO levels in Hep3B cells. Next, we transfected Hep3B cells with a p53-expressing plasmid (*TP53*), and p53 nuclear sequence deleted plasmid (*TP53*-ΔNLS). Compared to the empty vector-transfected control cells, the LPO level was significantly increased in *TP53*-transfected Hep3B cells but not in *TP53*-ΔNLS-transfected Hep3B cells (Figure 3E). Moreover, Sora-induced cell death was observed in *TP53* plasmid-transfected Hep3B cells but not in control or *TP53*-ΔNLS-transfected Hep3B cells (Figure 3F). We also transfected HepG2 cells with *TP53* and *TP53*-ΔNLS plasmids and found that Sora-induced LPO accumulation was only observed in *TP53* plasmid-transfected HepG2 cells but not in *TP53*-ΔNLS-transfected HepG2 cells (Figure 3G). Coincidentally, Sora-induced cell death was more obvious in *TP53* plasmids-transfected HepG2 cells (Figure 3H). The above evidence suggested that mitochondrial p53 retrograding into the nucleus initiated the LPO phenomenon and promoted Sora-induced ferroptosis, but the upstream mechanism remained unclear.

### Mitochondrial p-GPX4^Ser2^ dephosphorylation triggered ferroptosis and induced mitochondrial p53 translocation

**Figure 4:**
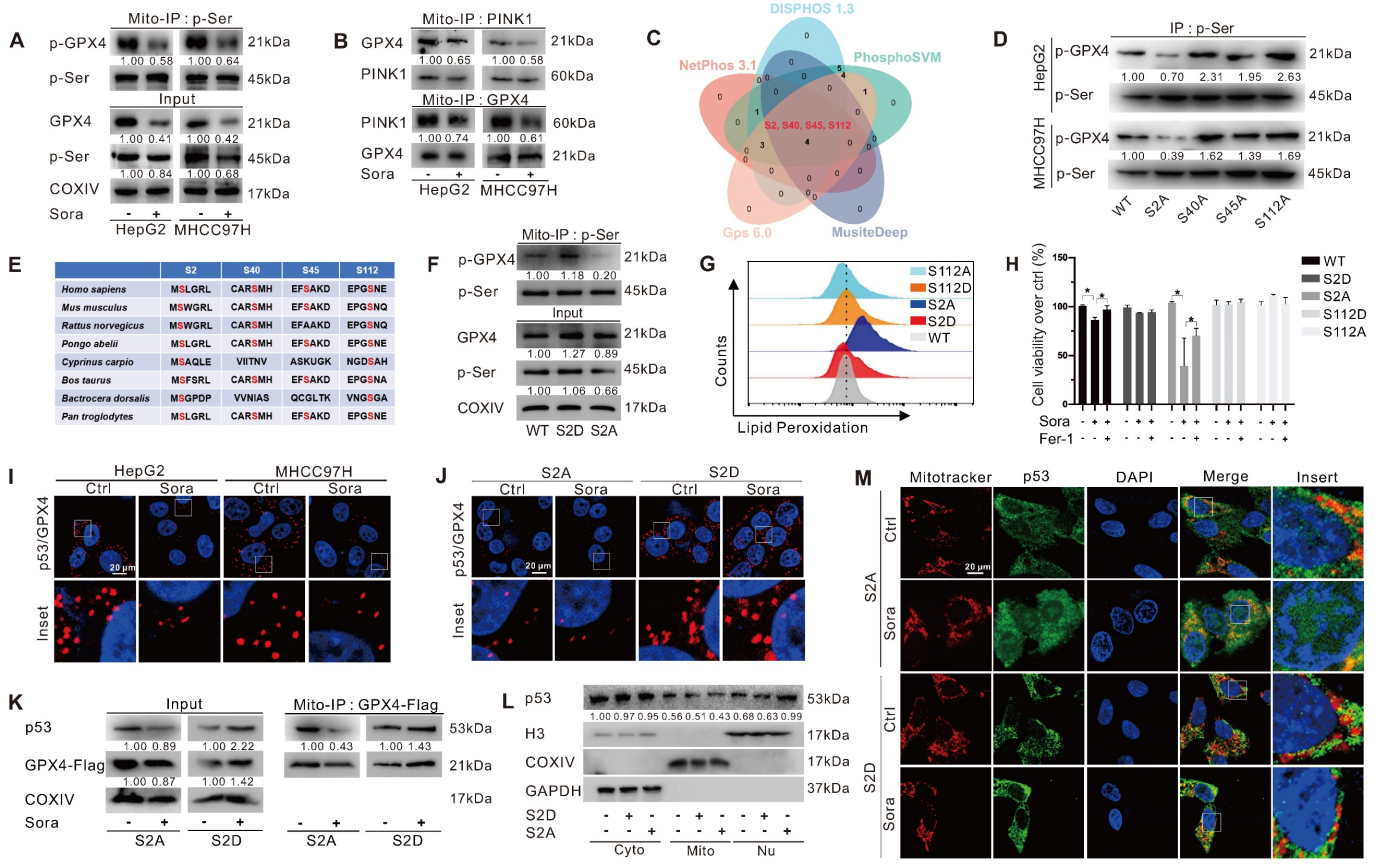
** Mitochondrial p-GPX4^Ser2^ dephosphorylation triggered ferroptosis and induced mitochondrial p53 translocation. A.** Mitochondrial-IP (Mito-IP) detection of the mitochondrial p-GPX4 level in HCC cells upon Sora treatment (10 µM, 24 h). **B.** Mito-IP detection of the interaction between GPX4 and PINK1 in mitochondria of HCC cells treated with Sora (10 µM, 24 h). **C.** Venn diagram showed the shared and unique phosphorylation sites of GPX4 predicated by DISPHOS 1.3, PhosphoSVM, MusiteDeep, Gps 6.0, and NetPhon3.1.** D.** IP detection of the phosphorylation levels of GPX4 in the constructed S2A, S40A, S45A, and S112A cells of HepG2. **E.** Sequences alignment of the conserved serine residues on GPX4. **F.** Mito-IP detection of the mitochondrial p-GPX4 levels in WT cells and the constructed S2D and S2A cells of HepG2.** G.** FCM detection of the LPO levels in WT, S2D, S112D, S2A, and S112A cells of HepG2. Staining cells with C11 BODIPY probe. **H.** WT, S2D, S112D, S2A, and S112A cells of HepG2 were treated with Sora (10 µM) individually or combined with Fer-1 (1 µM, 24 h). Cell viability was measured with the MTS assay.** I.** PLA detection of the interaction between p53 and GPX4 in HCC cells upon Sora treatment (10 µM, 24 h). **J.** PLA detection of the interaction between p53 and GPX4 in S2A and S112A cells of HepG2. **K.** Mito-IP detection of the interaction between GPX4 and p53 in S2D and S2A cells of HepG2 treated with Sora (10 µM, 24 h). **L**. Protein levels of p53 in cytosolic (Cyto), mitochondrial (Mito), and nucleus (Nu) fraction of WT, S2D, and S2A cells of HepG2. **M.** IF assay of the distribution of p53 in mitochondria and nucleus of S2D and S2A cells of HepG2 upon Sora treatment (10 µM, 24 h). Cells were subjected to IF staining of p53 (green), mitotracker (red), and DAPI (blue). Scale bars, 20 μm. *, *P* < 0.05.

GPX4 PTM plays a vital role in ferroptosis regulation. To explore whether the phosphorylation status of mitochondrial GPX4 was involved in Sora-induced ferroptosis, we examined the level of phosphorylated GPX4 (p-GPX4) in mitochondria of HCC cells upon Sora treatment. The results showed that the Sora treatment decreased the mitochondrial p-GPX4 level of HCC cells (Figure 4A). Next, we screened the potential kinases responsible for the phosphorylation of mitochondrial GPX4. As shown in Figure S2A and Figure 4B, among several common kinases, we found that Sora treatment decreased the protein level of PINK 1 and its interaction with mitochondrial GPX4 in HCC cells. Meanwhile, PINK1 knockdown decreased the level of p-GPX4 in HepG2 cells (Figure S2B), indicating that PINK1 might be one of the kinases responsible for mitochondrial GPX4 phosphorylation. Further, using 5 online phosphorylation prediction tools, we identified that the Ser2, Ser40, Ser45, and Ser112 sites were the unique and potential phosphorylation sites of GPX4 (Figure 4C), and only the Ser2 site mutation decreased the mitochondrial p-GPX4 level in HCC cells (Figure 4D), suggesting that the Ser2 site was one of the functional regulatory sites associated with GPX4 phosphorylation.

As Ser2 and Ser112 sites were highly conserved (Figure 4E), we explored the functions of the regulated Ser2 site phosphorylation in ferroptosis, and the Ser112 site was selected as a control. We mutated Ser2 and Ser112 sites of GPX4 to alanine (A) or aspartate (D) to generate GPX4 mimic dephosphorylation (S2A, S112A) or phosphorylation (S2D, S112D) cell lines of HepG2. As expected, the p-GPX4 level in mitochondria was increased in S2D cells, whereas it was reduced in S2A cells compared to that in WT cells (Figure 4F). Distinct from the ferroptosis resistance effects observed in HepG2 cells overexpressing WT and the other mutations of GPX4, Sora-induced LPO accumulation (Figure 4G) and cell death (Figure 4H) were only observed in S2A cells, suggesting that p-GPX4^Ser2^ dephosphorylation accelerated ferroptosis in HCC.

We next explored whether p-GPX4^Ser2^ dephosphorylation affected mitochondrial p53 translocation. As shown in Figure 4I, Sora treatment decreased the interaction between mitochondrial p53 and GPX4. Moreover, upon Sora treatment, a decreased interaction between mitochondrial p53 and GPX4 was only observed in S2A cells but not in S2D cells (Figure 4J-K). Further, upon Sora treatment, decreased mitochondrial p53 and increased nuclear p53 were displayed in S2A cells but not in S2D cells (Figure 4L-M and Figure S2C), implying that mitochondrial p-GPX4 dephosphorylation at the Ser2 site promoted the p53 translocation from mitochondria to the nucleus.

### PP2A-B55β regulated p-GPX4^Ser2^ dephosphorylation and promoted Sora-induced ferroptosis in HCC

**Figure 5:**
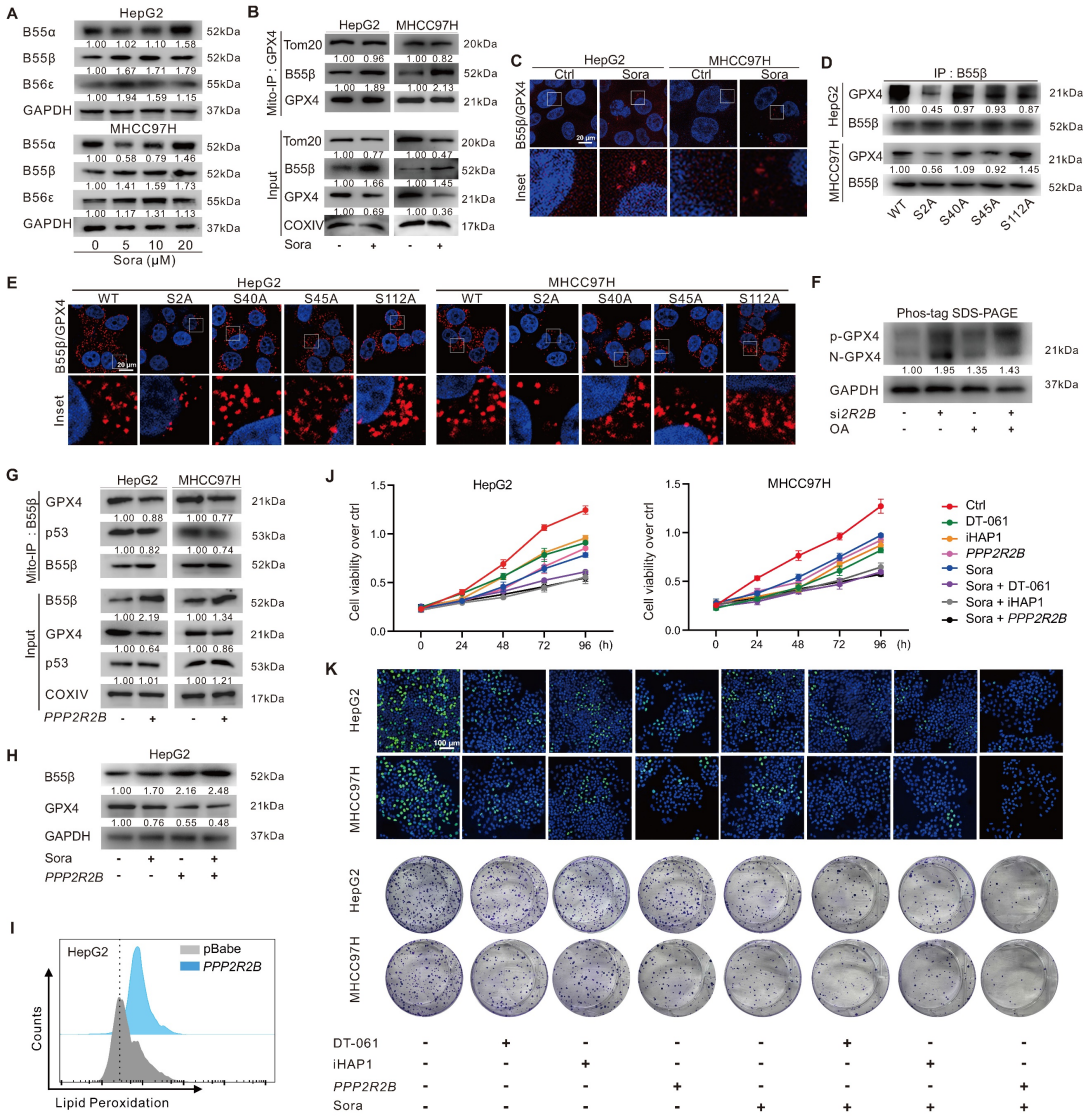
** PP2A-B55β regulated p-GPX4^Ser2^ dephosphorylation and promoted Sora-induced ferroptosis in HCC cells. A.** Protein levels of B55α (coded by *PPP2R2A*), B55β (coded by* PPP2R2B*), and B55ε (coded by* PPP2R5E*) in HCC cells treated with Sora (5, 10, 20 µM, 24 h). **B.** Mito-IP detection of the interaction between GPX4 and B55β in HCC cells treated with Sora (10 µM, 24 h). **C.** PLA detection of the interaction between B55β and GPX4 in HCC cells treated with Sora (10 µM, 24 h). Interaction events were shown as red dots. Scale bars, 20 μm.** D.** IP detection of the interaction between B55β and GPX4 in WT cells and the constructed S2A, S40A, S45A, and S112A cells.** E.** PLA detection of the interaction between B55β and GPX4 in WT, S2A, S40A, S45A, and S112A cells. Interaction events were shown as red dots. Scale bars, 20 μm. **F.** HepG2 cells were treated with si*2R2B* and/or OA, while the whole cell lysates were isolated and subjected to PhosTag™ gel electrophoresis. The protein levels of the phosphorylated GPX4 (p-GPX4) and non-phosphorylated GPX4 (N-GPX4) were tested. **G.** Mito-IP detection of the interaction between GPX4 and p53 in HCC cells transfected with the *PPP2R2B*-overexpression plasmid. **H.** Protein levels of B55β and GPX4 in HepG2 cells treated with *PPP2R2B*-overexpression plasmid and/or Sora (10 µM, 24 h). **I.** FCM detection of LPO levels in HepG2 cells transfected with the *PPP2R2B*-overexpression plasmid. Staining cells with C11 BODIPY probe.** J.** Cell viability of HCC cells treated with Sora (10 µM) alone or combined with *PPP2R2B*-overexpression plasmid or PP2A agonists DT-061 (20 µM) and iHAP1 (10 µM) for 24, 48, 72, and 96 h. **K**. EdU staining and colony formation assay detection of the cell proliferation ability of HCC cells treated with Sora (10 µM) alone or combined with *PPP2R2B*-overexpression plasmid or PP2A agonists DT-061 (20 µM) and iHAP1 (10 µM) for 24 h. *, *P* < 0.05.

Protein phosphatase 2A (PP2A), a serine/threonine phosphatase, regulates the activity of numerous enzymes and proteins via dephosphorylation modification 23-27. To investigate whether PP2A participated in regulating p-GPX4^Ser2^ dephosphorylation, we first analyzed the expression of the substrate recognition subunit of PP2A in HCC patients. As shown in Figure S3A, the levels of *PPP2R2A*,* PPP2R2B*, and *PPP2R2D* genes expression in tumor tissues of HCC patients from the GEO (GSE76427) and TCGA-LIHC dataset were lower than those in peritumor tissues. Further, we explored the relationship between the expression of PP2A subunits and GPX4 in HCC patients. The analysis revealed a negative correlation between* GPX4* and* PPP2R2A*,* PPP2R2B*, or *PPP2R5E* expression in HCC patients from the TCGA-LIHC dataset (Figure S3B). HCC patients with high *PPP2R2B* gene expression had a higher overall survival rate (Figure S3C). Moreover, upon Sora treatment, the protein level of PP2A-B55β (coded by *PPP2R2B*) was dose-dependent increased in HCC cells (Figure 5A). These results suggested that *PPP2R2B* might be against the development of HCC via the negative modulating of GPX4.

Next, the interaction between PP2A-B55β and GPX4 was explored. Molecular docking showed potential binding ability (Etotal = -591.4 kcal/mol, Eshape = -724.6 kcal/mol, Eforce = 133.2 kcal/mol) between PP2A-B55β and GPX4 (Figure S4A). The Mito-IP assay revealed that PP2A-B55β could interact with GPX4 on mitochondria, and the interaction was further enhanced upon treatment of Sora (Figure 5B). The IF method and proximity ligation assay (PLA) showed the increased PP2A-B55β and GPX4 interaction in HCC cells upon Sora treatment (Figure S4B and Figure 5C). Moreover, decreased PP2A-B55β and GPX4 interaction was only observed in S2A cells but not in S40A, S45A, or S112A cells (Figure 5D-E and Figure S4C), indicating that the Ser2 site of GPX4 played a crucial role in the regulation of PP2A-B55β-targeted GPX4 dephosphorylation. Additionally, PP2A-B55β knockdown (si*2R2B*) or PP2A inhibition (okadaic acid, OA, an inhibitor of PP2A) increased the protein level of p-GPX4 (Figure 5F), suggesting that PP2A-B55β was the upstream modulator responsible for the dephosphorylation regulation of p-GPX4.

Further, we explored whether PP2A-B55β could affect the p53 retrograde signaling and ferroptosis in HCC cells. As shown in Figure 5G, genetic (*PPP2R2B*) overexpression of B55β reduced the interaction between GPX4 and p53 on mitochondria. Meanwhile, B55β overexpression accelerated the GPX4 reduction (Figure 5H) and LPO accumulation (Figure 5I) in HepG2 cells. Moreover, using MTS assay, EdU assay, and colony formation assay, we found that the cell proliferation ability of HCC cells was decreased under Sora treatment, and the effects were further aggravated by the co-treatment of *PPP2R2B* overexpression plasmid or PP2A agonists DT-061 and iHAP1 (Figure 5J-K), indicating that PP2A-B55β activation enhanced the inhibitory effect of Sora on the proliferation ability of HCC cells. Our data revealed that PP2A-B55β might regulate the dephosphorylation of p-GPX4^Ser2^ and promote Sora-induced ferroptosis in HCC cells.

### PP2A-B55β promoted the anti-tumor effect of Sora via aggravation of ferroptosis

**Figure 6:**
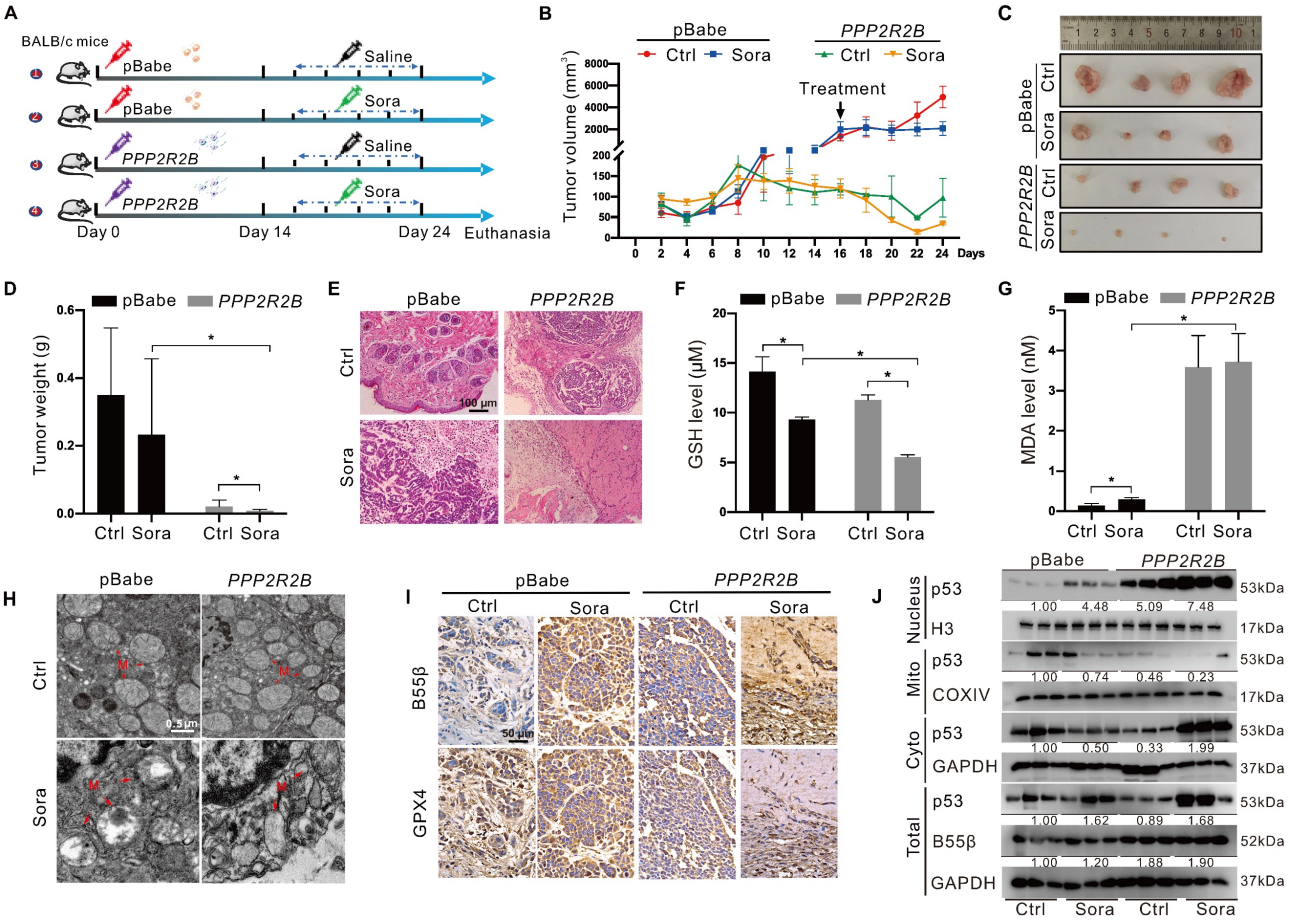
** PP2A-B55β promoted the anti-tumor effect of Sora via aggravation of ferroptosis. A.** The diagram depicted the experimental design of the *in vivo* study. BALB/c nude mice were subcutaneously inoculated with the constructed HepG2-*PPP2R2B* cells with B55β-overexpression, while HepG2-pBabe cells were used as control cells. Mice harbored with HCC-xenograft tumors were randomly divided into 4 groups (n = 4 for each group): pBabe-Ctrl, pBabe-Sora,* PPP2R2B*-Ctrl, *PPP2R2B*-Sora. The Sora (10 mg/kg) was injected via the tail vein every two days. **B.** The growth curves of xenograft tumors in nude mice. Tumor volume = (W^2^ × L)/2, where L is the longer dimension and W is the shorter one. **C-D.** Representative images (C) and weight (D) of xenograft tumors. **E.** Representative H&E staining images of xenograft tumor tissues. **F-G.** Levels of GSH (F) and MDA (G) in xenograft tumor tissues. **H.** TEM observation of mitochondrial morphological characteristics (red arrows) of HCC cells in xenograft tumor tissues. N, nucleus; M, mitochondria. Scale bar, 0.5 μm. **I.** Representative IHC staining images of GPX4 and B55β expression in xenograft tumor tissues. **J.** Protein levels of p53 in the nucleus and in the mitochondrial or cytoplasmic fractions of xenograft tumor tissues. *, *P* < 0.05.

Using the xenograft tumor assay, we explored the synergistic anti-tumor effects of genetic PP2A-B55β upregulation and Sora administration (Figure 6A). Xenograft tumor results of 24-day growth curves (Figure 6B), sizes (Figure 6C), and weight (Figure 6D) revealed that tumor growth of the B55β-overexpressing HCC cells was significantly suppressed while compared to the xenograft tumors with control cells. Moreover, compared to the obvious tumor-like mass lesions observed in the control xenograft tumors, B55β-overexpressing tumors were loosely arranged, and necrosis occurred (Figure 6E). These changes were aggravated under treatment with Sora, suggesting that PP2A-B55β expression in HCC enhanced the tumor-suppressive effect of Sora.

Further, we detected the effect of PP2A-B55β on the occurrence of ferroptosis in the xenograft tumors*.* Compared to the control tumors, reduced GSH and increased MDA were observed in the *PPP2R2B* overexpression tumors (Figure 6F-G). Meanwhile, TEM showed that the mitochondria became smaller in the *PPP2R2B* overexpression tumors, while the mitochondrial cristae disappeared, a characteristic change of ferroptosis (Figure 6H). Moreover, immunohistochemistry (IHC) analysis of the dissected xenograft tissues showed that GPX4 expression level was decreased in *PPP2R2B* overexpression tumors (Figure 6I). These changes were aggravated under the treatment of Sora, indicating that PP2A-B55β promoted Sora-induced ferroptosis in the HCC xenograft tumors. Then, we explored the effects of PP2A-B55β on the retrograde signal of mitochondrial p53 in the xenograft tumors. Consistent with the results *in vitro*, genetic *PPP2R2B* overexpression increased the protein level of nucleus p53 but decreased the protein level of mitochondrial p53 (Figure 6J). Altogether, these results obtained from the xenograft animal model suggested that PP2A-B55β enhanced Sora-induced ferroptosis, thereby inhibiting tumor growth.

## Discussion

HCC is the most common type of liver cancer with high morbidity and lethality, and its incidence continues to increase worldwide 28. Though Sora has shown significant effects in mitigating the development of HCC, drug resistance and poor prognosis to Sora therapy remain formidable challenges in human HCC.

The toxic effect of Sora on cancer cells partly relies on inducing ferroptosis. GPX4 is the only enzyme that can diminish lipid hydroperoxides of biological membranes, which is vital in protecting cells from oxidative damage in ferroptosis 4. Tumor cells may develop resistance to ferroptosis-dependent cancer treatment by augmenting GPX4 expression, and GPX4-targeted attenuation of its activity has been proposed as a potential approach to sensitize HCC to the treatment of Sora 29. In the present study, we found that a novel route enhanced the anti-tumor effect of Sora by affecting the mitochondrial GPX4 PTM. GPX4 (S2 site) Ser-to-Ala mutant (S2A), which mimics GPX4 dephosphorylation, failed to reverse the LPO generation in HCC cells and sensitized HCC to Sora-induced ferroptosis. Similar to us, the stable expression of GPX4 Sec-to-Ser mutant in the GPX4 deficiency cell model did not reverse cell death from lack of GPX4 30. A previous study reported that, compared to the Ala-to-Cys mutant of GPX4, the Ala-to-Sec mutant contributed to GPX4 binding of RSL3, a selective ferroptosis activator for tumor cells carrying oncogenic RAS 19. Furthermore, Michael et al. found that covalently modifying the Cys93 site of GPX4 by fumarates that accumulated in fumarate hydratase inactivation conditions promoted ferroptosis in renal carcinoma cells, highlighted a crucial role of GPX4 PTM in ferroptosis regulation 31. The current results and those of the previous studies suggested that mitochondrial GPX4 PTM might be an available strategy for regulatory ferroptosis in HCC. Our study firstly revealed that the dephosphorylation of mitochondrial GPX4 positively modulated ferroptosis in HCC.

Given the positive role of p-GPX4^Ser2^ dephosphorylation in Sora-induced ferroptosis, combining protein phosphatase activation and Sora may exert more effective anti-cancer effects in HCC treatment. PP2A is a major member of serine/threonine protein phosphatase family and is vital in mitochondrial quality control and cancer development 23-27. In response to endogenous and exogenous stimulation, the multiple regulatory B subunits of PP2A can be selectively translocated to mitochondria and are involved in various mitochondria-mediated physiopathological processes 24-27. Herein, we verified that PP2A-B55β not only directly interacted with mitochondrial GPX4 but might affect the stability of GPX4 by regulating the phosphorylation status of GPX4. Furthermore, PP2A-B55β overexpression facilitated Sora-induced GPX4 reduction and LPO accumulation in HCC cells and xenograft tumors *in vitro* and *in vivo*. The above evidences suggested that PP2A-B55β-mediated dephosphorylation of the p-GPX4 might result in the degradation of GPX4 and promote the occurrence of ferroptosis. The related research on PP2A and ferroptosis was very limited. FTY720, an FDA-approved immunosuppressant, showed obvious anti-tumor properties in many cancer models by inducing PP2A activation 32. Studies recently further verified that PP2A activation was essential for FTY720-induced ferroptosis in multiple myeloma cells, highlighting the role of PP2A in ferroptosis-associated cancer development 33. Although some pharmacological inhibitors (such as statins) could sensitize cancer cells to ferroptosis via targeted inhibition of GPX4, oxidative damage to normal cells and newly developed drug resistance restrict its clinical application 8, 34. The combined use of PP2A-B55β activation and Sora might be a promising strategy for enhancing ferroptosis-based HCC therapy.

Another novel finding of the present study was that GPX4 could interact with p53 in mitochondria under hyperphosphorylation status. Moreover, dephosphorylation of p-GPX4^Ser2^ decreased the GPX4 and p53 interaction and caused an obvious reduction of mitochondrial p53. p53 plays a vital role in the nucleus to mitochondrial communication. p53 translocation from the nucleus to mitochondria was proven to maintain mitochondrial homeostasis via interaction with multiple mitochondrial proteins 35. Furthermore, inconsistent with the dynamics of p53 in mitochondria, p-GPX4^Ser2^ dephosphorylation increased the p53 level in the nucleus, indicating that p-GPX4^Ser2^ dephosphorylation enhanced the p53 translocation from mitochondria to the nucleus (known as the retrograde signal of p53). The retrograde signal of p53 is an essential feedback approach for mitochondria-to-nucleus interorganellar communication. However, an excessive retrograde signal can lead to malignant cell fate 36. Herein, we found that the increased nucleus signal of p53 aggravated the occurrence of ferroptosis in HCC, whereas the phenomenon was not obvious in p53 nucleus deletion cells. Consistent with us, rotenone exposure triggered the retrograde signal of p53, and the signals preceded mitochondrion dysfunction and cell death 17. p53 has been verified to be a positive regulator in promoting ferroptosis, and multiple ferroptosis-related genes and proteins were proved to directly interact with p53 or be the transcriptional targets of p53 37. Therefore, the retrograde signal of p53 might be an early event of ferroptosis, which was one of underlying mechanisms for elevating ferroptosis via the regulation of p-GPX4^Ser2^ dephosphorylation.

We also noted that there were some limitations of the present study. First, since the phosphorylation modification of the Ser2 site of GPX4 was not confirmed by LC/MS/MS analysis in HEK293T cells, the functional screening and regulation of p-GPX4^Ser2^ in Sora-treated HCC cells should be concerned with caution and remained for further validation. Moreover, the present study suggested that PINK1 might be the potential kinase responsible for mitochondrial GPX4 phosphorylation, which would be another research direction that remains verified by the *in vitro* phosphorylation and IP analysis, as well as for the targeted intervention of GPX4 phosphorylation. Nevertheless, the present study identified that the B55β/p-GPX4^Ser2^/p53 axis was a novel regulatory pathway of ferroptosis. Mitochondrial p-GPX4^Ser2^ dephosphorylation initiated ferroptosis in HCC via promoting the retrograde signaling of p53. The targeting regulation of PP2A-B55β on the dephosphorylation of p-GPX4^Ser2^ could serve as a novel targeted intervention strategy for enhancing ferroptosis-dependent tumor theranostics.

## Methods

### Bioinformatics analysis

Public datasets, including the GEO database (GSE102079) and the Cancer Genome Atlas (TCGA) liver hepatocellular carcinoma (LIHC) dataset, were used to extract RNA expression data from HCC patients. We utilized LIMMA to conduct the differentially expressed genes (DEGs) analysis. The Log2 (fold change) and *P*-value were 1.0 and 0.05, respectively. The DEGs analysis and Gene Ontology (GO) enrichment were performed on R studio software (version 4.1.1). Gene set enrichment analysis (GSEA) was performed using the GSEA software (version 4.1.0) to analyze the enrichment of the ferroptosis pathway in tumors. Expression and survival analysis of GPX4 in LIHC were conducted using the GEPIA database (http://gepia.cancer-pku.cn/index.html).

### HCC tissue specimens

HCC tumor tissues and the corresponding peritumor tissues were obtained from six HCC patients at Xiang'an Hospital of Xiamen University (Xiamen, China). HCC tissue specimens were immediately frozen in liquid nitrogen and then stored at -80 °C for the follow-up experiments. Part of the HCC tissue specimens were fixed and paraffin-embedded before the pathological examination. Western blotting (WB) and immunohistochemistry (IHC) staining were used to detect the protein levels of p53 and GPX4. All patients signed the informed consent. The present study was approved by the Ethics Committee of Xiamen University and performed following the Helsinki Declaration.

### *In vivo* xenograft tumor study

Six-week-old BALB/c nude mice were purchased from SLAC Laboratory Animal Co. Ltd (Shanghai, China). HepG2-*PPP2R2B* cells with B55β-overexpression were constructed, while HepG2-pBabe cells were used as control cells. 3 × 10^6^ cells (HepG2-pBabe or HepG2-*PPP2R2B*) in 50 μL PBS combined with 50 μL of Matrigel (BD, CA, USA) were subcutaneously inoculated into the right flank of each mouse. When xenograft tumors reached palpable size (at day 14 after injection), inoculated mice were randomly divided into 4 groups (n = 4): pBabe-Ctrl, pBabe-Sora,* PPP2R2B*-Ctrl, *PPP2R2B*-Sora. The saline or Sora (10 mg/kg) was injected via the tail vein every two days. The dimensions of xenograft tumors were measured using a digital caliper. The formula (W^2^ × L)/2 was used to estimate tumor volume, where L is the longer dimension and W is the shorter one. After five 2-day cycles of treatment, the mice were executed and xenograft tumors were excised and weighed. Xenograft tumor tissues were collected for subsequent Western blotting (WB) or tissue sectioning and IHC analysis. All experiments were approved by the Experimental Animal Ethics Committee of Xiamen University (Ethic protocol code: XMULAC20180094).

### IHC analysis and hematoxylin-eosin (HE) staining

The dissected xenograft tumor tissues were fixed in a 4% paraformaldehyde (PFA) fix solution, followed by paraffin embedding and serially sectioning at 4 μm thickness. Sections were deparaffinized with xylene and dehydrated with an ethanol series of increasing concentrations. IHC detection was accomplished with the UltraSensitive^TM^ SP IHC Kit (MXB, Fuzhou, China) as previously described 38. Information on the antibodies, including anti-B55β, anti-GPX4, and anti-p53, were listed in Table S1. Sections were subsequently stained with HE for 10 min. Then, the sections were washed with xylene after dehydration in gradient ethanol solutions, sealed with neutral resin, and observed by an inverted microscope.

### Detection of malondialdehyde (MDA) and glutathione (GSH) levels

Xenograft tumor tissues (about 5 mg) were washed with pre-cooled PBS. 200 μL of PBS was added to prepare tissue homogenate, followed by the dilution with saline and subsequent determination. According to the manufacturer's instructions, MDA and GSH levels were tested using the corresponding reagent kits (Nanjing Jiancheng, China). Optical density values were detected by the microplate reader, while the excitation wavelength was 530 nm for MDA and 405 nm for GSH. Protein concentrations were tested using bicinchoninic acid (BCA) protein assay to calculate MDA and GSH levels per mg of protein.

### Cell culture and reagents

HepG2, MHCC97H, Hep3B, and HEK293T cell lines were stocked in our laboratory. Cells are maintained in Dulbecco's Modified Eagle Medium (DMEM, Gibco, NY, USA) with 10% fetal bovine serum (FBS, Gibco, CA, USA) and 1% penicillin-streptomycin (Gibco, CA, USA) at 37 °C in a 5% CO_2_ humidified incubator (Thermo, CO, USA). Dimethyl sulfoxide (DMSO) was purchased from Sigma (MO, USA). Sorafenib (Sora) and Ferrostatin-1 (Fer-1) were purchased from Selleck (TX, USA).

### Establishment of GPX4-overexpressing and its phosphorylated site-directed mutagenesis cells

The construction of stable GPX4-overexpressing HepG2-*GPX4* cells was the same as our previously described 38. In brief, full-length *GPX4* coding sequence was obtained and incorporated into the retroviral vector pBabe-puro to construct the pBabe-*GPX4* recombinant plasmid. HEK293T cells were co-transfected with retroviral plasmid and pCL-Ampho vector in a ratio of 1:1. HepG2 cells were transfected with retroviruses produced by HEK293T cells. The established cell lines were screened with 0.6 μg/mL puromycin. The phosphorylation sites of GPX4 were predicated by DISPHOS 1.3 (http://www.dabi.temple.edu/disphos/), PhosphoSVM (http://sysbio.unl.edu/PhosphoSVM/), MusiteDeep (https://www.musite.net/), Gps 6.0 (http://gps.biocuckoo.cn/), and NetPhon3.1 (https://services.healthtech.dtu.dk/services/NetPhos-3.1/). The serine 2 (S2), serine 40 (S40), serine 45 (S45), and serine 112 (S112) sites of GPX4 were mutated to alanine (A) or aspartate (D) according to the instructions of the KOD-Plus Mutagenesis Kit (Toyobo, Osaka, Japan). Information of mutation primers was shown in Table S2.

### Cell viability assay

Cell viability assay was performed as previously described 39. In brief, 1 × 10^4^ cells of each cell line were seeded on 96-well plates and treated with different administration. MTS solution was added and the cells were incubated for 3 h at 37 °C. The absorbance at 490 nm was quantified using a microplate spectrophotometer system (Multiskan, Thermo, USA). Fer-1 and inhibitors of different cell death patterns were used in combination with Sora to identify whether cell viability was affected by ferroptosis.

### Transmission electron microscope (TEM) analysis

After treatment with 10 µM of Sora for 24 h, the cells were collected, fixed with 0.1 M glutaraldehyde buffer, post-fixed with 4% PFA, dehydrated with ethanol and propylene oxide, and finally embedded in epoxy resin. About 70-80 nm thickness sections were sliced and stained with uranyl acetate and lead citrate. Mitochondrial and nuclear ultrastructure was evaluated using a TEM (Tecnai 20, FEI, USA). Similarly, for animal study, xenograft tumor tissues were collected and followed the above methods.

### Immunofluorescence (IF) analysis

Cells were mounted on coverslips for different experimental conditions and stained with 100 nM MitoTracker Red CMXRos (Life, CA, USA) in a culture medium at 37 °C for 30 min. Cells were fixed in 4% PFA and permeabilized with 0.5% Triton X-100 in PBS for 5 min. After blocked in PBS containing 1% BSA, cells were incubated overnight with primary antibodies (anti-B55β, anti-GPX4, or anti-p53), followed by the appropriate fluorescent secondary antibodies for 1 h in the dark. Detail information on the antibodies was listed in Table S1. Experiments for detecting protein changes in the nucleus need to be stained with DAPI for 5 min before viewing and shooting. Cells were viewed using a laser-scanning confocal microscope (Leica SP8, Wetzlar, Germany).

### Proximity ligation assay (PLA)

The protein-protein interaction studies were performed with PLA. According to the manufacturer's instructions, a Duolink® *In Situ* Detection Reagent (Sigma, MO, USA) was used. Briefly, cells were mounted on sterile coverslips in 24-well plates at a density of 5 × 10^4^ cells per well overnight. After being fixed with 4% PFA and permeabilized using 0.5% Triton X-100, cells were blocked in Duolink II solution for 1 h. The coverslips were incubated with anti-p53, anti-B55β, or anti-GPX4 at 4 °C overnight (the detailed information on the antibodies was listed in Table S1), followed by Duolink PLA anti-Rabbit PLUS and PLA anti-Mouse PLUS proximity probes. After washing the coverslips three times, the ligation reaction was conducted for 30 min, and the amplification was run for 100 min at 37 °C. Then the coverslips were visualized using a confocal microscope (Leica SP8, Wetzlar, Germany). Images were analyzed with IPP 6.0 software.

### Lipid peroxidation (LPO) assay

Cells were seeded into 6-well plates with 3×10^5^ per well. After the indicated treatment, the cells were digested and stained with 2 μM BODIPY-C11 (Invitrogen, CA, USA) for 40 min at 37 °C, followed by flow cytometry analysis (Beckman, CA, USA). At least 1×10^4^ cells were analyzed for each condition, each experiment was independently performed at least 3 times, and representative experimental results were shown. Data analysis was performed using the FlowJo 10.4 software.

### Isolation of mitochondrial, nuclear, and cytosolic fractions

Mitochondrial and cytosolic fractions were prepared using a mitochondrial isolation kit (Enzo Life, PA, USA). Briefly, 5×10^7^ cells of each group were harvested and centrifuged at 600 × *g* at 4 °C for 5 min. The pellets were resuspended in mitochondria isolation buffer and centrifuged at 600 × *g* for 10 min. The supernatants were collected and centrifuged at 12,000 × *g* for 10 min. The pellet was mitochondria, and the supernatant was the cytosolic fraction. Resuspended the pellet in mitochondria lysate buffer and centrifuged at 12,000 × *g* at 4 °C for 10 min to achieve mitochondrial fractions. Proteins were quantified with the BCA protein assay and were used for Western blotting (WB) analysis.

As for the nuclear fraction, cells were harvested and centrifuged at 600 × *g* for 4 min at 4 °C to remove the supernatant. Cells were resuspended with PMSF-added cytoplasmic protein extraction reagent A followed by the ice bath for 15 min. Then cytoplasmic protein extraction reagent B was added and centrifuged at 16,000 × *g* at 4 °C for 5 min. The supernatant was a cytoplasmic fraction. After sucking up the supernatant, the nuclear protein extraction reagent containing PMSF was added and centrifuged at 16,000 × *g* for 10 min at 4 °C. The supernatant is the nuclear fraction.

### Western blotting (WB) analysis and immunoprecipitation (IP) assay

Cells were washed with ice-cold PBS, and whole-cell lysates were prepared in an SDS/β-mercaptoethanol sample buffer containing protease inhibitors. Proteins were separated by 10-12% SDS-PAGE gels, transferred to PVDF membranes (Millipore, MA, USA), blocked in 5% skimmed milk for 1 h at room temperature then incubated with the primary antibodies at 4 °C overnight. After incubation with goat Anti-Rabbit-IgG-HRP or goat Anti-Mouse-IgG-HRP for 1 h at room temperature, proteins were visualized by enhanced chemiluminescence. IP assay was performed as described previously 40. The detailed information on the primary antibodies including the manufacturers, code number, the dilution used in WB, IP, and IHC, molecule weight, and species were listed in Table S1.

### Quantitative real-time polymerase chain reaction (qRT-PCR)

Total RNA was extracted from cells using TRIzol reagent (Ambion, TX, USA). cDNA was reversed from total RNA using PrimeScript^TM^ RT reagent kit (TaKaRa, Otsu, Japan) and qRT-PCR was subsequently performed the same in our previous study 38. The relative mRNA transcription levels of genes were displayed using the 2^-ΔΔCt^ method. The information of primers used in this study was listed in Table S2.

### Statistics

Statistical analyses were performed using the Statistical Package for Social Sciences (SPSS) version 16.0 (SPSS, Chicago, IL, USA). Unless indicated, all data are shown as mean ± standard deviation (SD) of at least three independent experiments. Statistical comparison of mean values in two groups was assessed using the Student's *t*-test. Multiple groups were compared by one-way analysis of variance (ANOVA) with Dunnett's post-test. Pearson's correlation analysis was performed for the correlation between variables. A *P*-value of < 0.05 was considered to be statistically significant. All the experiments were repeated at least three times.

## Supplementary Material

Supplementary figures and tables.

## Abbreviations

Ala: alanine
Asp: aspartate
BCA: bicinchoninic acid
IP: immunoprecipitation
Cyto: cytosolic
DEGs: differentially expressed genes
FCM: flow cytometry
GEO: gene expression omnibus
GO: gene ontology
GPX4: glutathione peroxidase 4
GPX4^ser2^: GPX4 serine 2
GSEA: Gene set enrichment analysis
GSH: glutathione
HCC: hepatocellular carcinoma
H3: Histone 3
IHC: immunohistochemistry
IF: immunofluorescence
LIHC: liver hepatocellular carcinoma
LPO: lipid peroxidation
MDA: malondialdehyde
Mito: mitochondrial
MMP: mitochondrial membrane potential
OMM: outer mitochondrial membrane
PLA: proximity ligation assay
PP2A: phosphatase protein phosphatase 2A
PTM: post-translational modification
qRT-PCR: quantitative real-time polymerase chain reaction
ROS: reactive oxygen species
Ser: serine
Sora: sorafenib
System Xc-: cystine-glutamate antiporter
TCGA: the cancer genome atlas
TEM: transmission electron microscope
WT: wild type
WB: western blotting
