# Supplementary material for: Protein phosphatase 2A-B55β mediated mitochondrial p-GPX4 dephosphorylation promoted sorafenib-induced ferroptosis in hepatocellular carcinoma via regulating p53 retrograde signaling
Source: Theranostics. 2023 Jul 31;13(12):4288–302. doi: 10.7150/thno.82132 (PMC10405852; doi:10.7150/thno.82132)
Supplement: Supplementary file 1 — Supplementary figures and tables. [file thnov13p4288s1.pdf]

# Protein phosphatase 2A-B55β mediated mitochondrial p-GPX4 dephosphorylation promoted sorafenib-induced ferroptosis in hepatocellular carcinoma via regulating p53 retrograde signaling

Bo Qian [1†], Lin Che [1†], Ze-Bang Du [1†], Ni-Jun Guo [1†], Xin-Mou Wu [1], Lei Yang [1], Zhao-Xuan Zheng [1], Yun-Lu Gao [1], Ming-Zhu Wang [1], Xiao-Xuan Chen [1], Ling Xu [1], Zi-Jian Zhou [1,2*], Yu-Chun Lin [1*], Zhong-Ning Lin [1*]

[1] State Key Laboratory of Vaccines for Infectious Diseases, Xiang An Biomedicine Laboratory; State Key Laboratory of Molecular Vaccinology and Molecular Diagnostics; National Innovation Platform for Industry-Education Integration in Vaccine Research; School of Public Health, Xiamen University, Xiamen, China.

[2] Center for Molecular Imaging and Translational Medicine, School of Public Health, Xiamen University, Xiamen, China.

[†] These authors contributed equally.

* Corresponding author: zhouz@xmu.edu.cn (Z.-J. Zhou), linych@xmu.edu.cn (Y.-C. Lin), and linzhn@xmu.edu.cn (Z.-N. Lin)

## Supplementary Materials

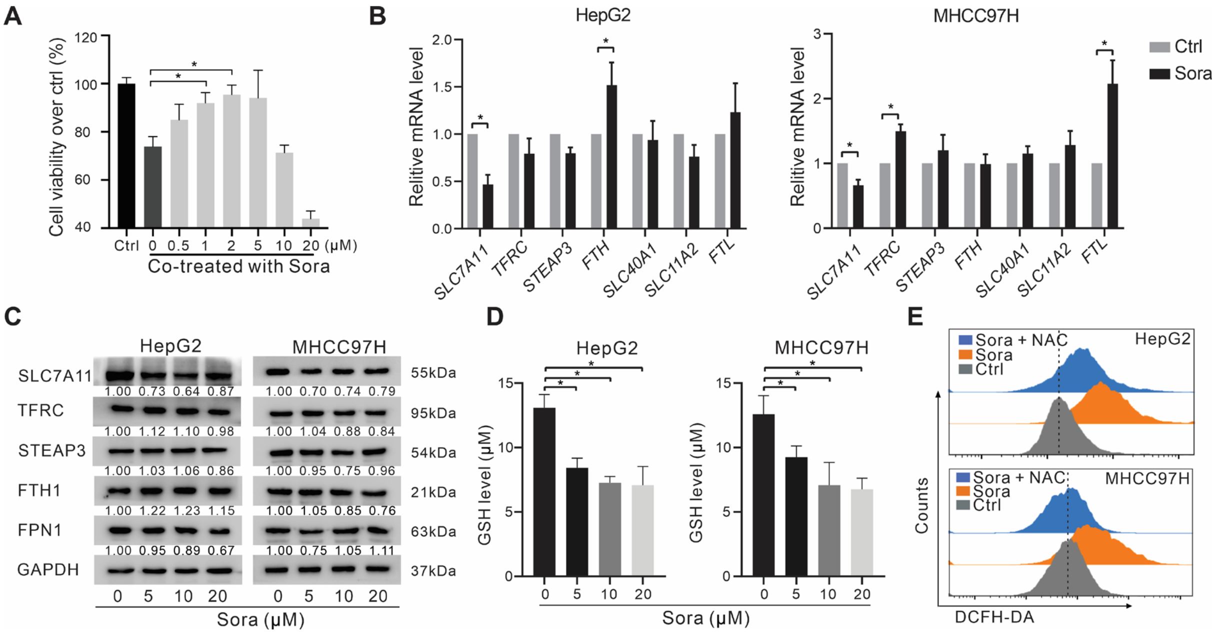


**Figure S1. Sorafenib induced GPX4-related ferroptosis in HCC cells. A.** HepG2 cells were treated with sorafenib (Sora, 10 μM) and co-treated with different concentrations of the ferroptosis inhibitor Ferrostatin-1 (Fer-1, 1 μM) for 24 h. Cell viability was measured with the MTS assay. **B.** The mRNA levels of *SLC7A11*, *TFRC*, *STEAP3*, *FTH*, *SLC40A1*, *SLC11A2*, and *FTL* in HCC cells upon Sora treatment (10 μM, 24 h). **C.** The protein levels of SLC7A11, TFRC, STEAP3, FTH1, and FPN1 in HCC cells upon Sora treatment (5, 10, 20 μM, 24 h). **D.** GSH level in HCC cells upon Sora treatment (5, 10, 20 μM, 24 h). **E.** The ROS level in Sora-treated HCC cells. HCC cells were individually treated with Sora (10 μM, 24 h) or co-treated with ROS inhibitor NAC (10 μM, 12 h). Cells was stained with a DCFH-DA fluorescent probe and tested with FCM. * $P < 0.05$.

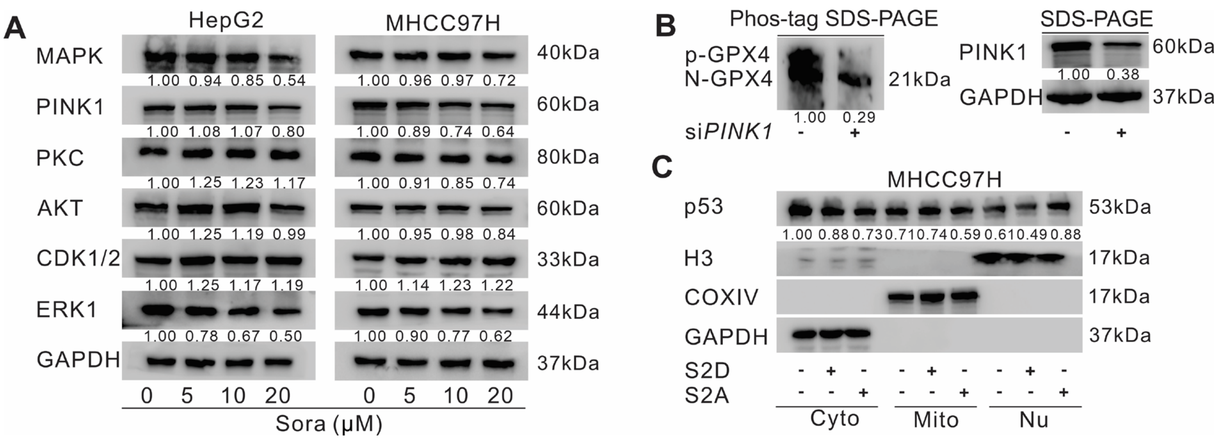


**Figure S2. Dephosphorylation of mitochondrial p-GPX4$^{Ser2}$ triggered ferroptosis and induced mitochondrial p53 translocation. A.** Protein levels of MAPK, PINK1, PKC, AKT, CDK, and ERK in HCC cells treated with Sora (5, 10, 20 µM, 24 h). **B.** HepG2 cells were treated with si*PINK1* (50 nM, 24 h). Whole cell lysates were isolated and subjected to Phos-tag SDS-PAGE, and the protein levels of the phosphorylated GPX4 (p-GPX4) and non-phosphorylated GPX4 (N-GPX4) were tested. **C.** Protein levels of p53 in cytosolic (Cyto), mitochondrial (Mito), and nucleus (Nu) fractions of WT cells and the constructed S2D or S2A MHCC97H cells, n = 3.

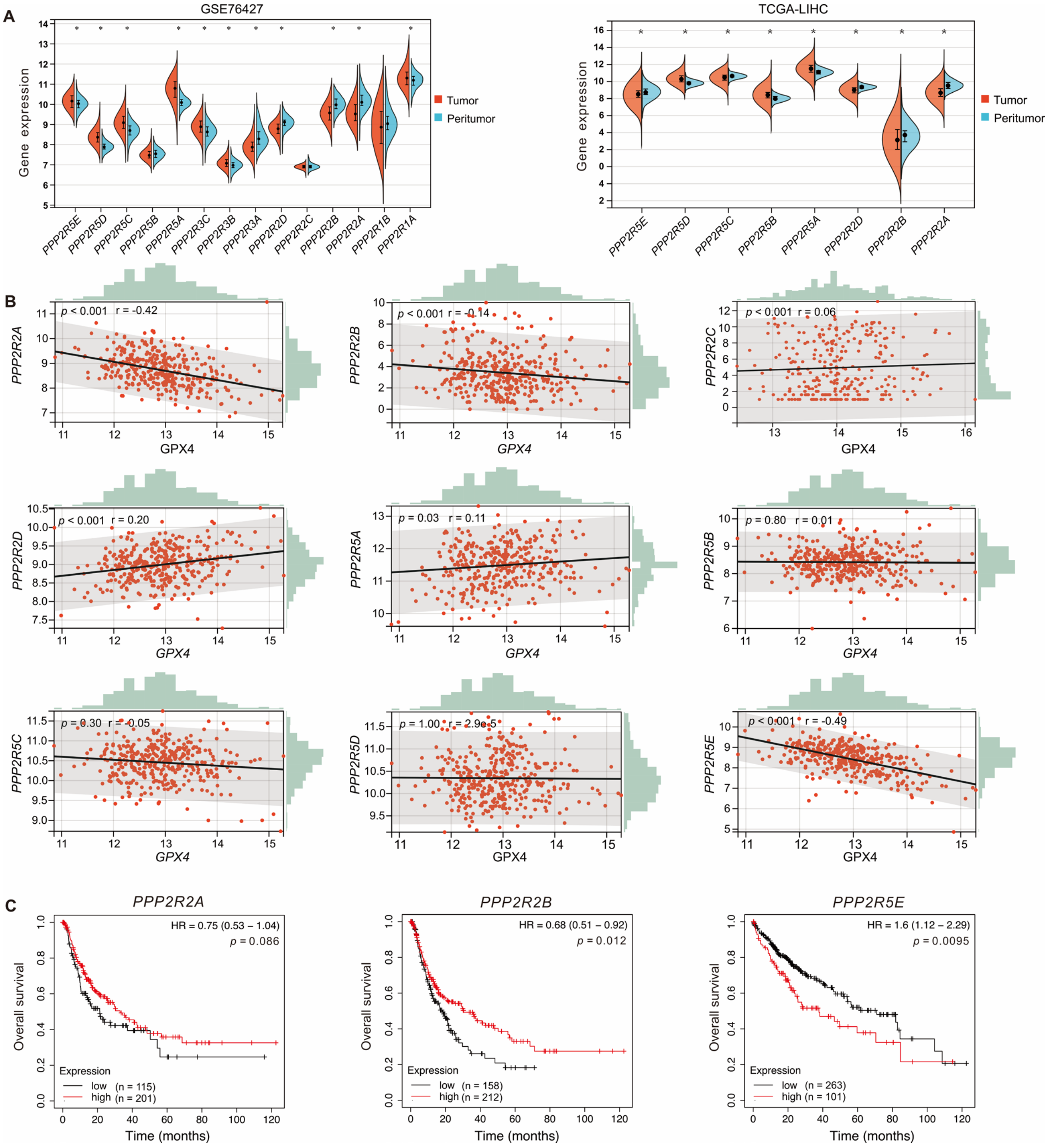


**Figure S3. *PPP2R2B* associated with the development of HCC via the negative modulating on GPX4 expression. A.** The expression of various regulatory B subunits of PP2A in tumor tissues and peritumor tissues of HCC patients from the GEO database (GSE76427, n = 243) and TCGA-LIHC database (n = 423). **B.** The correlation between the expression of *GPX4* and regulatory B subunits of PP2A in HCC patients from the TCGA-LIHC database (n = 423). **C.** Kaplan-Meier analysis showed the overall survival of HCC patients from TCGA-LIHC with different expressions (low or high level) of *PPP2R2A*, *PPP2R2B*, and *PPP2R5E*.

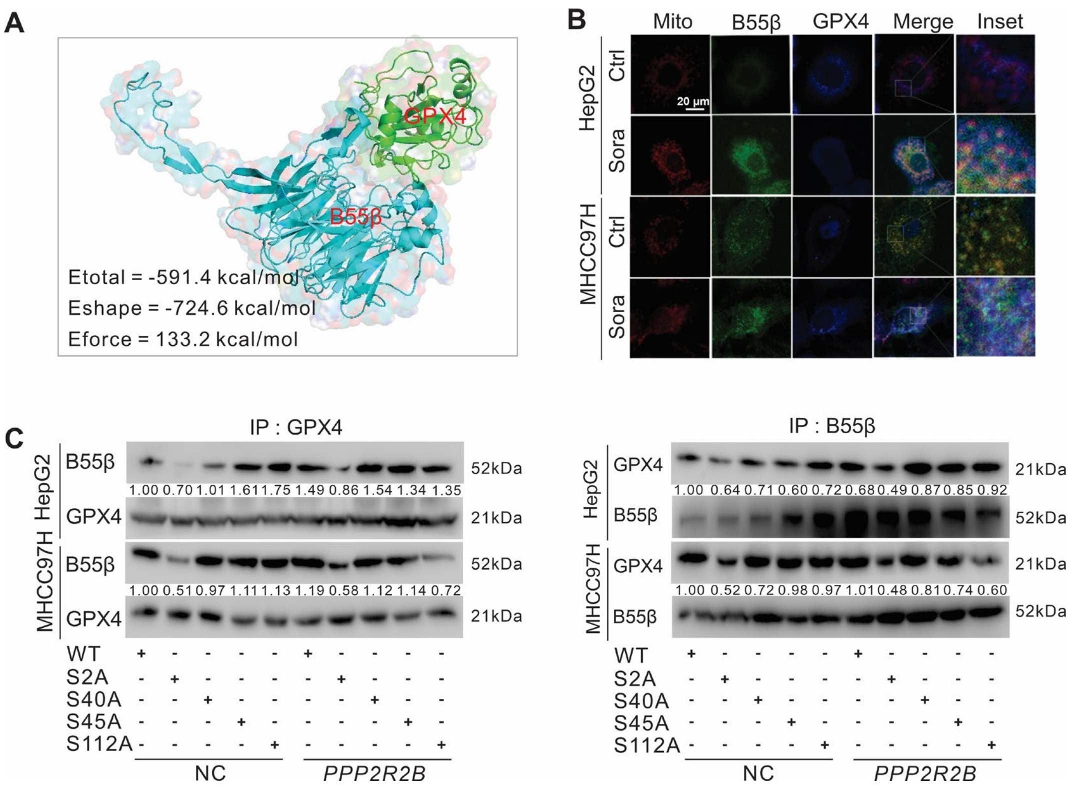


**Figure S4. PP2A-B55β interacted with GPX4 and potentially targeted dephosphorylation regulation.** **A.** Molecular docking between GPX4 and B55β was performed using the Hex Protein Docking software (Hex 8.0.0). **B.** The distribution of B55β and GPX4 in mitochondria of HCC cells treated with Sora (10 μM, 24 h). Scale bars, 20 μm. **C.** HCC cells (HepG2 and MHCC97H cells) were transfected with the pBabe-*PPP2R2B* plasmid to construct the B55β-overexpression cells following site-directed mutation of the serine (S) 2, S40, S45, and S112 sites of GPX4, while S to A (alanine) mimics the dephosphorylation. The pBabe plasmid-transfected cells were as negative control (NC). The potential target proteins in whole cell lysates were pulled down by GPX4 (Left) and B55β (Right) antibodies. The interaction between B55β and GPX4 was detected by Co-IP.

**Table S1. Information on the primary antibodies used in the present study**

| Primary antibodies | Manufacturers | Code number | Dilution/Concentration | | | | Molecule weight | Species |
|---|---|---|---|---|---|---|---|---|
| | | | WB | IF/PLA | IP | IHC | | |
| GPX4 | Santa Cruz | sc-166570 | 1:400 | 1:100 | 5 μg | 1:100 | 21 kDa | Mouse |
| p53 | Abcam | ab26 | 1:1000 | 1:400 | 5 μg | | 53 kDa | Mouse |
| B55α | Abcam | ab197194 | 1:1000 | | | | 52 kDa | Rabbit |
| B55β | Abcam | ab251885 | 1:1000 | 1:400 | 5 μg | 1:200 | 52 kDa | Rabbit |
| B56ε | Abcam | ab198290 | 1:1000 | | | | 55 kDa | Rabbit |
| Phosphoserine | IMMUNECHEM | ICP9806 | 1:250 | | 5 μg | | 45 kDa | Rabbit |
| FLAG | HUABIO | HA601080 | 1:5000 | | 5 μg | | 35 kDa | Mouse |
| Histone 3(H3) | Cell Signaling Technology | #4499 | 1:2000 | | | | 17 kDa | Rabbit |
| COXIV | Abcam | ab16056 | 1:2000 | | | | 17 kDa | Rabbit |
| GAPDH | LABLEAD | G0100-100UL | 1:5000 | | | | 37 kDa | Mouse |
| TOM20 | Santa Cruz | sc-17764 | 1:400 | | | | 20 kDa | Mouse |
| SLC7A11 | Abcam | ab307601 | 1:1000 | | | | 55 kDa | Rabbit |
| FPN1 | Santa Cruz | sc-518125 | 1:400 | | | | 63 kDa | Mouse |
| STEAP3 | Abcam | ab151566 | 1:1000 | | | | 54 kDa | Rabbit |
| TFRC | Santa Cruz | sc-393719 | 1:300 | | | | 95 kDa | Mouse |
| FTH1 | Cell Signaling Technology | #4393 | 1:1000 | | | | 21 kDa | Rabbit |
| MAPK | Cell Signaling Technology | #8690 | 1:1000 | | | | 40 kDa | Rabbit |
| PINK1 | Cell Signaling Technology | #6946 | 1:1000 | | | | 60 kDa | Rabbit |
| PKC | Santa Cruz | sc-17769 | 1:400 | | | | 80 kDa | Mouse |
| AKT | Cell Signaling Technology | #4685 | 1:1000 | | | | 60 kDa | Rabbit |
| CDK1/ CDK2 (CDK1/2) | Santa Cruz | sc-53219 | 1:400 | | | | 33 kDa | Mouse |
| ERK1 | Cell Signaling Technology | #4372 | 1:1000 | | | | 44 kDa | Rabbit |

**Table S2. Information of the paired primers in the present study**

| Experiment | Genes | Primers | Sequences (5'- 3') |
|---|---|---|---|
| **Real-time PCR** | *SLC7A11* | FP | TCCTGCTTTGGCTCCATGAACG |
| | | RP | AGAGGAGTGTGCTTGCGGACAT |
| | *SLC40A1* | FP | TGAGCCTCCCAAACCGCTTCCATA |
| | | RP | GGGCAAAAAGACTACAACGACGACTT |
| | *STEAP3* | FP | TGCAAACTCGCTCAACTGGAGG |
| | | RP | AGGCAGGTAGAACTTGTAGCGG |
| | *SLC11A2* | FP | AGCCACTCAGGTATCCACCAT |
| | | RP | CCAGGGGACTGTGAAAGAGAG |
| | *FTH1* | FP | TGAAGCTGCAGAACCAACGAGG |
| | | RP | GCACACTCCATTGCATTCAGCC |
| | *FTL* | FP | AGCCTTCTTTGTGCGGTCGGGTAA |
| | | RP | ACGCCTTCCAGAGCCACATCAT |
| | *TFRC* | FP | ACCATTGTCATATACCCGGTTCA |
| | | RP | CAATAGCCCAAGTAGCCAATCAT |
| **Site-directed mutagenesis** | S2A | FP | ATGGCCCTCGGCCGCCTTTGCCGCCTACTG |
| | | RP | GAGCAGCGCCGGCTTCAGTAGGCGGCAAAG |
| | S2D | FP | ATGGATCTCGGCCGCCTTTGCCGCCTACTG |
| | | RP | GAGCAGCGCCGGCTTCAGTAGGCGGCAAAG |
| | S40A | FP | GCCATGCACGAGTTTTCCGCCAAGGACATC |
| | | RP | AACCATGTGCCCGTCGATGTCCTTGGCGGA |
| | S45A | FP | GCCGCCAAGGACATCGACGGGCACATGGTT |
| | | RP | GTACTTGTCCAGGTTAACCATGTGCCCGTC |
| | S112A | FP | GCCAACGAAGAGATCAAAGAGTTCGCCGCG |
| | | RP | TTTGACGTTGTAGCCCGCGGCGAACTCTTT |
| | S112D | FP | GATAACGAAGAGATCAAAGAGTTCGCCGCG |
| | | RP | TTTGACGTTGTAGCCCGCGGCGAACTCTTT |
